# A community-based prospective cohort study of exclusive breastfeeding in central Nepal

**DOI:** 10.1186/1471-2458-14-927

**Published:** 2014-09-08

**Authors:** Rajendra Karkee, Andy H Lee, Vishnu Khanal, Colin W Binns

**Affiliations:** School of Public Health and Community Medicine, BP Koirala Institute of Health Sciences, Dharan, Nepal; School of Public Health, Curtin University, GPO Box U1987, Perth, WA 6845 Australia; Sanjeevani College of Medical Sciences, Butwal, Rupandehi, Nepal

**Keywords:** Exclusive breastfeeding, Risk factors, Nepal

## Abstract

**Background:**

Existing information on breastfeeding in low income countries such as Nepal has been largely derived from cross-sectional demographic health surveys. This study investigated exclusive breastfeeding rates, and compared the duration of exclusive breastfeeding between rural and urban mothers in central Nepal using an alternate cohort methodology.

**Methods:**

A community-based prospective cohort study was conducted among 639 recently delivered mothers representative of the Kaski district of Nepal. Breastfeeding information was obtained at birth (n = 639), 4 weeks (n = 639), 12 weeks (n = 615; 96.2%) and 22 weeks (n = 515; 80.6%) through repeated interviews using validated questionnaires. Risk of cessation of exclusive breastfeeding was assessed by Cox regression analysis.

**Results:**

The great majority of women received breastfeeding information (74%) and were encouraged to breastfeed by health personnel or family members (81%). Although nearly all mothers (98%) breastfed up to six months, the reported exclusive breastfeeding rate declined rapidly from 90.9% at birth to 29.7% at 22 weeks. Urban women experienced significantly shorter (p = 0.02) exclusive breastfeeding duration (mean 104.5, 95% CI 95.8 to 113.1 days) and were more likely to cease exclusive breastfeeding (hazard ratio (HR) 1.28, 95% CI 1.03 to 1.60) than their rural counterparts (mean 144.7, 95% CI 132.3 to 157.1 days). Breastfeeding problem (HR 2.07, 95% CI 1.66 to 2.57) and caesarean delivery (HR 1.88, 95% CI 1.36 to 2.62) were also significantly associated with exclusive breastfeeding cessation.

**Conclusions:**

Despite the almost universal practice of breastfeeding, the reported exclusive breastfeeding rates declined substantially over time. Exclusive breastfeeding up to six months was more common in rural than urban areas of central Nepal. Urban mothers also exclusively breastfed shorter than rural mothers.

**Electronic supplementary material:**

The online version of this article (doi:10.1186/1471-2458-14-927) contains supplementary material, which is available to authorized users.

## Background

Appropriate infant feeding, namely, early initiation of breastfeeding with colostrum as the first food, exclusive breastfeeding to six months, followed by the introduction of complementary foods with continue breastfeeding, is important for survival as well as physical growth and mental development of the child [1–3]. Infants who are not exclusively breastfed for the first six months have high rates of mortality and morbidity, particularly those associated with diarrhoea and pneumonia [4, 5]. Globally, 11.6% of child deaths in 2011 are due to suboptimal breastfeeding [6]. Moreover, breastfeeding is known to have long term health benefits against non-communicable diseases [7].

Despite efforts to improve breastfeeding rates over the past two decades, including implementation of the International Code of Marketing of Breastmilk Substitutes, the Baby-Friendly Hospital Initiative, and launching of the WHO/UNICEF’s Global Strategy for Infant and Young Child Feeding, the global rate of exclusive breastfeeding has remained stagnant below 40% [8, 9]. In South Asia, exclusive breastfeeding has increased slightly from 40% in 1995 to 45% in 2010 [10], but comparisons between surveys should be made with caution because different definitions and methodologies were used in calculating exclusive breastfeeding rates [11, 12]. Moreover, the rate of exclusive breastfeeding for the first six months varies in relation to maternal and environmental factors [13–15]. A study conducted in southern Nepal, for example, found that women from hill origin exclusively breastfed longer than women residing in Terai (plain area) [16]. Higher rates of breastfeeding and exclusive breastfeeding are generally observed among lower income countries, while rural mothers usually breastfeed longer than their urban counterparts [8].

In the literature, reports on breastfeeding in Nepal were mainly derived from National Demographic Health Surveys (NDHS) [17]. The NDHS collected retrospective cross-sectional information on infant feeding in the past 24-hours from mothers who gave birth within two years preceding the survey, which could incur recall bias when assessing exclusive breastfeeding. In addition, the data, often presented as a period prevalence rate, might be difficult to interpret. Several other studies conducted in Nepal also adopted a cross-sectional design and did not cover the entire first six months of life [18, 19]. In view of these shortcomings, a prospective cohort study of community-dwelling women was conducted to investigate exclusive breastfeeding rates, to compare the exclusive breastfeeding duration between rural and urban mothers, and to determine factors associated with early cessation of exclusive breastfeeding in central Nepal. We hypothesized that exclusive breastfeeding is different between rural and urban locations, with a higher risk of early cessation among urban women.

## Methods

### Study setting

Nepal is a small but diverse country in South Asia. Female literacy rate is 67% and total fertility rate is 2.6 births per women. The median age at first birth is 20.2 years. Most of the married women are occupied with home duties [20]. The location of this study was Kaski, a centrally located hills district of Nepal. The district has 75% female literacy rate and approximately 13,800 babies are born annually [21].

### Study design and participants

A large community-based prospective cohort study of utilisation of maternity services was conducted between December 2011 and November 2012 in the Kaski district. Details about participants, study design and sampling procedure had been described elsewhere [22]. Briefly, a total of 701 pregnant women of 5 months or more gestational age were recruited from five urban wards and seven rural *illakas* of the Kaski district, and were followed up for six months after delivery. Fifteen female data enumerators, who were recruited locally, searched for and identified pregnant women in their locality with the help of female community health volunteers and health facility registrations for antenatal care visits. They then visited the pregnant women’s houses.

The study was conducted according to the guidelines laid down in the Declaration of Helsinki and all procedures involving human subjects/patients were approved by the Human Research Ethics Committee of Curtin University (approval number HR 130/2011), and the Ethical Review Board of the Nepal Health Research Council (approval number 88/2011). A STROBE checklist can be found in Additional file 1. Written informed consent was obtained from all subjects. An information sheet was distributed and read to each consented subject before obtaining her signature or thumb-print. Confidentiality of the information provided was maintained throughout the study.

### Data collection

The questionnaire used in the interview was adapted from the NDHS and another validated instrument on breastfeeding; see Additional file 2 [23]. It was pretested on 25 postpartum women for cultural appropriateness, content validity and understanding. Fifteen female community health volunteers, trained by the first author, conducted the baseline interviews to collect information on socio-demographic and obstetric characteristics of the pregnant women. The participants were followed up with three subsequent household visits by the same female enumerators at 4 weeks, 12 weeks and 22 weeks postpartum, respectively. Breastfeeding at birth was assessed during the second follow-up interview.

### Statistical analysis

The outcome variable was the reported duration of ‘exclusive breastfeeding’, defined as the infant being given breastmilk only without any other feeds (aside from medications) since birth [2]. The main exposure variable of interest was residential location (rural versus urban). Table 1 lists other maternal socio-demographic and obstetric variables. Four levels of education were recorded, namely, none, primary (1-5^th^ grade), secondary (6-10^th^ grade), and college (after 10^th^ grade). Caste was defined according to the government’s classification in the health system. ‘Upper caste’ and ‘lower caste’ referred to Indo-Aryan people, whereas ‘janajati’ referred to Tibeto-Burman people and the term ‘religious minorities’ denoted people who are Muslim or Christian. In this study, only three women belonged to the religious minority so they were merged with the janajati group. Employment status was categorised as employed (full-time salaried job), semi-employed (wage based labour, small business or employed abroad), and unemployed (agricultural, housewife or nothing). Breastfeeding information was recorded yes if the woman had reported receiving any breastfeeding education or information from anywhere on how to feed her baby. Encouragement to breastfeed was considered present if the woman had reported receiving encouragement or motivation to breastfeed from health workers, family members or relatives. Breastfeeding problem meant painful swelling of breast, and inverted, cracked or sore nipples affecting lactation.

**Table 1:** **Maternal characteristics by residential location, Kaski District, Nepal (n = 639)**

Characteristic	Urban	Rural	Total	p*
	n (%)	n (%)	n (%)	
	343 (53.7)	296 (46.3)	639	
**Age** (years)				0.07
15 - 19	40 (11.7)	51 (17.2)	91 (14.2)	
20 - 24	173 (50.4)	151 (51.0)	324 (50.7)	
25 - 40	130 (37.9)	94 (31.8)	224 (35.1)	
**Parity**				0.29
Primiparous	170 (49.6)	159 (53.7)	329 (51.5)	
Multiparous	173 (50.4)	137 (46.3)	310 (48.5)	
**Employment status**				< 0.001
Unemployed	249 (72.6)	260 (87.8)	509 (79.7)	
Semi-employed	71 (20.7)	27 (9.1)	98 (15.3)	
Employed	23 (6.7)	9 (3.0)	32 (5.0)	
**Caste**				< 0.001
Upper caste	171 (50.3)	168 (56.8)	339 (53.3)	
Janajati	97 (28.5)	41 (13.9)	138 (21.7)	
Lower caste	72 (21.2)	87 (29.4)	159 (25.0)	
**Education**				< 0.001
None	31 (9.0)	22 (7.4)	53 (8.3)	
Primary	72 (21.0)	61 (20.6)	133 (20.8)	
Secondary	96 (28.0)	142 (48.0)	238 (37.2)	
College	144 (42.0)	71 (24.0)	215 (33.6)	
**Place of delivery**				< 0.001
Home	29 (8.5)	63 (21.4)	92 (14.5)	
Facility	311 (91.5)	231 (78.6)	542 (85.5)	
**Method of delivery**				< 0.001
Vaginal	277 (81.2)	273 (92.9)	550 (86.6)	
Caesarean	64 (18.8)	21 (7.1)	85 (13.4)	
**Frequency of antenatal visits**				0.67
< 4	92 (26.8)	75 (25.3)	167 (26.1)	
≥ 4	251 (73.2)	221 (74.7)	472 (73.9)	
**Breastfeeding information**				< 0.001
Yes	212 (61.8)	258 (87.2)	470 (73.6)	
No	131 (38.2)	38 (12.8)	169 (26.4)	
**Encouragement to breastfeed**				0.22
Yes	271 (79.0)	245 (82.8)	516 (80.8)	
No	72 (21.0)	51 (17.2)	123 (19.2)	
**Sex of infant**				0.61
Male	206 (60.1)	172 (58.1)	378 (59.2)	
Female	137 (39.9)	124 (41.9)	261 (40.8)	
**Breastfeeding problem**				0.001
No	244 (71.1)	172 (58.1)	416 (65.1)	
Yes	99 (28.9)	124 (41.9)	223 (34.9)	

*Chi-square test between urban and rural participants.

In the presence of censoring observations due to lost to follow up or continuation of exclusive breastfeeding beyond the study period, Cox’s proportional hazards modelling was used to assess the effect of residential location on the exclusive breastfeeding duration, accounting for plausible demographic and obstetric confounding variables. Both crude and adjusted hazards ratios (HR) and their 95% confidence intervals (CI) were reported for each significant factor to estimate the corresponding risk of cessation of exclusive breastfeeding, and backward stepwise analysis was undertaken in view of the apparent collinearity between exposure variables. All statistical analyses were performed using the SPSS package version 21.

## Results

### Characteristics of participants

**Figure 1:**
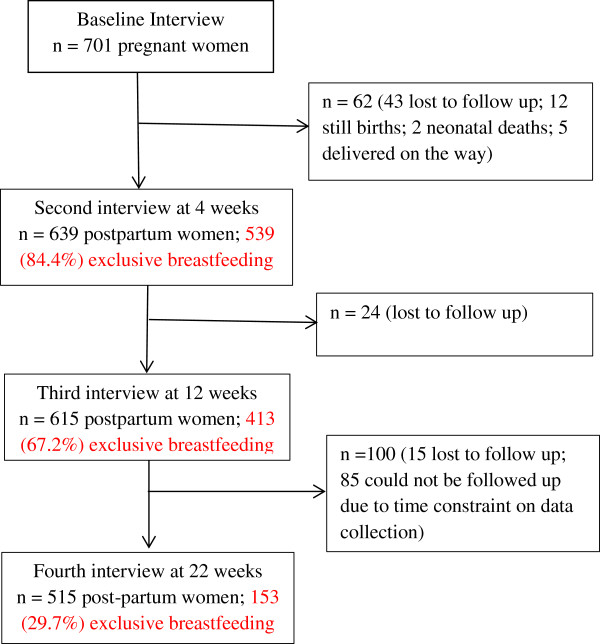
**Study flow chart.**

Figure 1 shows the flow chart of the cohort. Of the initial 701 pregnant women recruited, 639 (91.2%), 615 (87.7%), and 515 (73.5%) mothers were followed up at 4 weeks, 12 weeks, and 22 weeks after delivery, respectively. Table 1 presents the sample characteristics by residential location. About half of the mothers were primiparous (51.5%) within the age group 20–24 years (50.7%). The great majority of them were unemployed (79.7%), and had normal delivery (86.6%) at a health facility (85.5%). Almost all pregnant women (98%) had made at least one antenatal care visit. Most mothers reported receiving information about breastfeeding (73.6%) and were encouraged to breastfeed by health personnel or family members (80.8%), yet 35% of them experienced problems during lactation. Moreover, an examination using Spearman’s rho indicated that certain variables were highly correlated, e.g. caste and education, age and parity. When comparing urban versus rural mothers, the two groups appeared to be significantly different in terms of employment, caste, education, place of delivery, method of delivery, availability of breastfeeding information, and presence of breastfeeding problem; see Table 1. In particular, the prevalence of vaginal delivery was higher among rural women and more of them gave birth at home and received breastfeeding information relative to their urban counterparts.

### Exclusive breastfeeding rate and duration

Table 2 summarises reported breastfeeding rates at birth, 4, 12 and 22 weeks. Although nearly all women (98%) breastfed their infants up to six months, overall the exclusive breastfeeding rate declined from 90.9% at birth to 29.7% at 22 weeks. The main supplementary foods introduced were cow or buffalo milk while only a small proportion of the cohort (6% to 13%) fed their babies formula during the period. The data also indicated consistently higher rates of exclusive breastfeeding at birth and during follow up among rural mothers than urban mothers.

**Table 2:** **Breastfeeding status by residential location, Kaski district, Nepal, 2012**

Breastfeeding	At birth			4 weeks			12 weeks			22 weeks		
	Urban n = 343	Rural n = 296	Total n = 639	Urban n = 343	Rural n = 296	Total n = 639	Urban n = 319	Rural n = 296	Total n = 615	Urban n = 282	Rural n = 233	Total n = 515
	n (%)	n (%)	n (%)	n (%)	n (%)	n (%)	n (%)	n (%)	n (%)	n (%)	n (%)	n (%)
Any	297 (86.6)	284 (95.9)	581 (90.9)	339 (98.8)	296 (100.0)	635 (99.3)	311 (97.4)	296 (100)	607 (98.6)	275 (97.5)	230 (98.7)	505 (98.0)
Exclusive	297 (86.6)	284 (95.9)	581 (90.9)	280 (81.6)	259 (87.5)	539 (84.4)	201 (63.0)	212 (71.6)	413 (67.2)	76 (26.9)	77 (33.0)	153 (29.7)

Of the 639 women in the cohort, 581 mothers fed colostrum to their infants at birth and 58 mothers gave other liquid as the first feed. This group of 581 women was eligible for survival analysis of exclusive breastfeeding duration. Kaplan-Meier log-rank test showed significant difference in exclusive breastfeeding between rural and urban women (p = 0.02), with rural mothers (mean 144.7, 95% CI 132.3 to 157.1 days) exclusively breastfed longer than urban mothers (mean 104.5, 95% CI 95.8 to 113.1 days). Table 3 presents the result of stepwise Cox regression analysis, which confirm that urban women were more likely to cease exclusive breastfeeding (HR 1.28, 95% CI 1.03 to 1.60) after adjustment for confounders. Other significant risk factors associated with the discontinuation of exclusive breastfeeding were reported breastfeeding problem (HR 2.07, 95% CI 1.66 to 2.57) and caesarean delivery (HR 1.88, 95% CI 1.36 to 2.62). The Kaplan-Meier curves corresponding to these three factors are shown in; see Additional file 3: Figure S1.

**Table 3:** **Factors associated with cessation of exclusive breastfeeding, Kaski District, Nepal, 2012 (n = 581)**

Factor	Crude hazard ratio	Adjusted hazard ratio*	p
	(95% CI)	(95% CI)	
Residential location			0.02
Rural	1	1	
Urban	1.21 (0.98, 1.49)	1.28 (1.03, 1.60)	
Method of delivery			< 0.001
Vaginal	1	1	
Caesarean	2.12 (1.54, 2.93)	1.88 (1.36, 2.62)	
Breastfeeding problem			< 0.001
No	1	1	
Yes	2.03 (1.61, 2.47)	2.07 (1.66, 2.57)	

*Factors excluded from the backward stepwise Cox regression were maternal age, parity, employment status, caste, education, place of delivery, frequency of antenatal visits, breastfeeding information, encouragement to breastfeed, and sex of infant.

## Discussion

Despite almost all mothers breastfed their infants during the six months follow up, only 30% reported exclusive breastfeeding at 22 weeks postpartum. Since about 20% of the 639 participants were lost to follow up, the actual exclusive breastfeeding rate is likely to be lower. Breastfeeding is traditionally a common practice in Nepalese society, where most women have no salaried employment and are housewives, especially those residing in rural areas. Therefore, they have time to breastfeed their infants. Furthermore, women in the study district are highly aware of breastfeeding benefits, due to counselling during antenatal visits and after delivery [22]. Indeed, most women (74%) made at least four antenatal visits while facility delivery was also high particularly among urban participants (91.5%). Besides, support and encouragement can play an important role in breastfeeding duration [23–25]. About 80% of our mothers were encouraged to breastfeed by health workers or family members, which contributed to the reported high rate of breastfeeding in the study district.

The reported rate of exclusive breastfeeding declined substantially from birth to 22 weeks after delivery. Cow and/or buffalo milk is locally available and commonly regarded as alternative if breastmilk was perceived insufficient by the women. Moreover, in the Nepalese custom, almost all infants are likely to be introduced a solid food with a cultural ceremony called “pasni” by the end of six months, which might explain the decline in exclusive breastfeeding rates among both rural and urban mothers. In comparison, the recent NDHS reported that about 70% of infants under six months of age are exclusively breastfed in Nepal [20], whereas an urban cross-sectional study found prevalence of exclusive breastfeeding of 74%, 24%, and 9% at 1, 3, and 6 months, respectively [19]. The apparently higher rate from the NDHS survey might be caused by overestimation since the mothers’ recall of feeding history was limited to the preceding 24 hours at the time of interview [26]. Globally, exclusive breastfeeding rate for infants under six months, based on national demographic survey data, ranged from 1% in Djibouti to 85% in Rwanda [8].

Exclusive breastfeeding also tends to vary within a country. In Nepal, the recent NDHS survey reported that the exclusive breastfeeding rate is higher among illiterate and poor women living in rural areas [20]. A study in Bangladesh similarly found that urban region, high maternal education and high socio-economic status were risk factors for the discontinuation of exclusive breastfeeding [27]. Another cohort study in China showed that more rural mothers exclusively breastfed than urban mothers [28]. In Nepal, urban women are more likely to be partially or fully employed, accessible to infant formula as well as exposed to their advertisements [17], which tend to have negative impact on exclusive breastfeeding. The finding of this study that urban women discontinued exclusive breastfeeding earlier than rural women adds to this line of evidence.

In this study, almost 40% of urban women did not report receiving breastfeeding information and their prevalence of caesarean section was higher than women residing in rural areas. Breastfeeding problem and caesarean delivery are well established risk factors for the cessation of exclusive breastfeeding [13, 28, 29]. Breastfeeding problems such as inverted, cracked or sore nipples can impact on the lactation decision and practice, while caesarean section can affect the secretion of breastmilk as well as the general health condition of the mother to exclusively breastfeed. In this study, exclusive breastfeeding duration was not associated with other demographic and obstetric factors including maternal age, education and parity.

### Limitations

A major strength of the present study concerns the prospective assessment of breastfeeding practices from newly delivered women to minimise recall bias. However, all information obtained, including breastfeeding information and encouragement to breastfeed, was based on self-report by the participants. The occurrence of some recall error on breastfeeding practices and duration during the personal interview could not be ruled out, especially for breastfeeding at birth which was asked about 4 weeks later. Despite feeding status was assessed at periodic intervals, the response might not represent the actual feeding practice throughout the whole 6-month period, because some women merely reported what they were feeding around the time of each interview. This study did not specifically capture whether water was given to the infant. Water was not generally considered as a food so that some participants might perceive they were feeding only breastmilk even though water was given occasionally.

Loss to follow up poses as another limitation. Due to time limit and budgetary constraint, the exact duration of exclusive breastfeeding could not be recorded for all infants and follow up of participants were conducted at periodic intervals without covering the full 6 months (approximately 4, 12 and 22 weeks postpartum). Finally, the findings may not be generalised to the whole country as Nepal is diversified in terms of ecology, ethnicity and social development. The Kaski district was ranked third in human development index out of 75 districts in Nepal with high adult literacy (82%), and rural areas are connected to the central urban valley by gravelled roads [30].

## Conclusions

Exclusive breastfeeding up to six months was more common in rural than urban areas of central Nepal. Despite the almost universal practice of breastfeeding, the reported exclusive breastfeeding rates declined substantially over time. Moreover, the duration of exclusive breastfeeding was found to be significantly associated with residential location, caesarean delivery and the presence of breastfeeding problem. Exclusive breastfeeding for at least six months should be encouraged, along with support, encouragement and the provision of comprehensive breastfeeding information to minimise potential problems during lactation, which would lessen the risk of early cessation of exclusive breastfeeding, especially for urban mothers.

## Electronic supplementary material

Additional file 1: **STROBE Statement-Checklist of items that should be included in reports of** ***cohort studies***. (DOC 99 KB)

Additional file 2: **Questionnaire: Infant feeding information and practices.** Follow-up for infant feeding practices at 12 and 22 weeks after delivery. (DOCX 18 KB)

Additional file 3: Figure S1: Kaplan-Meier survival curves of cessation of exclusive breastfeeding by residential location, breastfeeding problem and delivery method, Kaski District, Nepal, 2012. (DOCX 53 KB)
