# Supplementary material for: A community-based prospective cohort study of exclusive breastfeeding in central Nepal
Source: BMC Public Health. 2014 Sep 8;14:927. doi: 10.1186/1471-2458-14-927 (PMC4161870; doi:10.1186/1471-2458-14-927)

Figure S1: Kaplan-Meier survival curves of cessation of exclusive breastfeeding by residential location, breastfeeding problem and delivery method, Kaski District, Nepal, 2012.


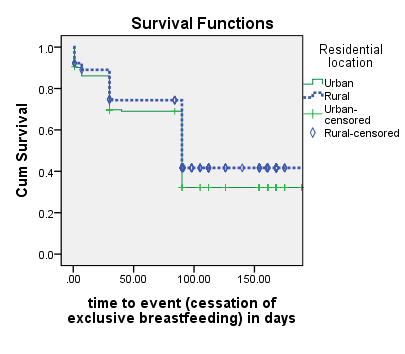

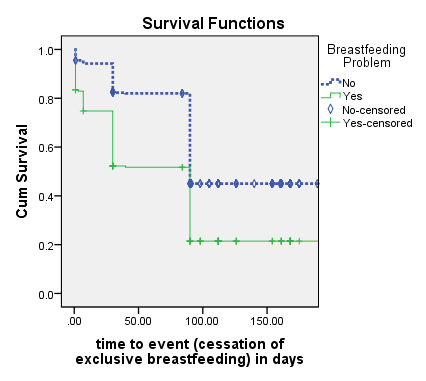

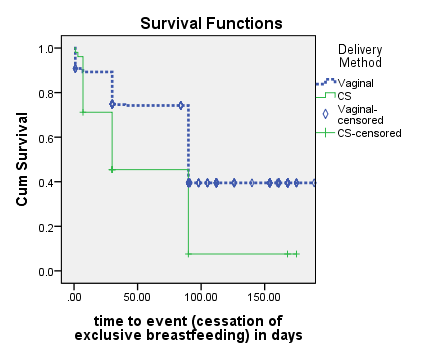

Supplement: Supplementary file 3 — Additional file 3: Figure S1: Kaplan-Meier survival curves of cessation of exclusive breastfeeding by residential location, breastfeeding problem and delivery method, Kaski District, Nepal, 2012. (DOCX 53 KB) [file 12889_2014_7023_MOESM3_ESM.docx]
